# The influence of strategic foresight on quality of healthcare services in the presence of artificial intelligence solutions in Jordan

**DOI:** 10.1186/s12912-024-02518-3

**Published:** 2025-02-13

**Authors:** Salma Sami Alajrab, Islam Ali Oweidat, Omaima Nassar, Mohammed ALBashtawy, Abdulqadir J. Nashwan

**Affiliations:** 1 Faculty of Nursing, Zarqa University, Zarqa, Jordan; 2 Community and Mental Health Nursing Department, Faculty of Nursing, Zarqa University, Zarqa, Jordan; 3 Department of Accreditation, Healthcare Accreditation Council, Amman, Jordan; 4 Princess Salma Faculty of Nursing, Al al-Bayt University, Mafraq, Jordan; 5 Nursing and Midwifery Research Department, Hamad Medical Corporation, P. O. Box 3050, Doha, Qatar

**Keywords:** Artificial intelligence (AI) based solutions, Strategic foresight, Quality of healthcare services, Nurses

## Abstract

**Background:**

Healthcare organizations are distinguished by intricate systems that undergo continual modifications and unpredictability. This greatly hinders the ability to estimate the exact consequences of any changes accurately. Therefore, scholars prove that strategic foresight enables leaders to anticipate future challenges and possibilities. The utilization of artificial intelligence (AI) in management is on the rise, mostly because of its ability to provide intelligent services, reduce medical errors, and improve operational efficiency.

**Purpose:**

To examine the impact of strategic foresight on the quality of healthcare services provided by Jordanian nurses in the context of AI solutions in governmental hospitals.

**Method:**

A cross-sectional descriptive correlational analysis was conducted. A convenience sampling approach was used in the four selected Jordanian governmental hospitals. The study’s target population consisted of nurses. Over three weeks between January and February 2024, 240 self-reported questionnaires were received using a five-point Likert scale, with a response rate of 88.9%. The completed surveys were suitable for analysis using AMOS SPSS v. 26 and SPSS.

**Results:**

Simple linear regression and (Pearson’s r) test results showed that (*R* = .279, R square = 0.078) between strategic foresight and the quality of healthcare services. (*R* = .543, R square = 0.295) between strategic foresight and the adoption of AI-based solutions. And (*R* = .432, R square = 0.187) between adopting AI-based solutions and the quality of healthcare services. That reveals a statistically significant, positive correlation coefficient relationship between the variables. In the presence of the mediator, the direct relationship between strategic foresight and healthcare service quality was not statistically significant (b = 0.063, *p* = .398). The path analysis test indicates a linear relationship between the variables sequentially, and the AI-based solutions completely mediate the relationship between strategic foresight and the quality of healthcare services.

**Conclusions:**

A positive and significant correlation between the variables suggests that a simulation-proposed model for a healthcare quality forecasting system, which the researcher built and included in the study recommendations, has to be designed. Therefore, AI-based forecasting systems should incorporate health service quality parameters to facilitate high efficiency and prompt patient demand fulfillment.

## Background

Experts agree that AI-based solutions offer significant advantages, including automation of manual tasks, improved data understanding, objective data analysis, trend identification, strategic uncertainty reduction, and improved decision-making [1]. AI-based medical and digital health solutions enhance daily lives and improve diagnosis, patient care, and healthcare research and development [2]. Digital health integrates digital technology and health information to improve patient health and healthcare delivery effectiveness [3, 4]. Digital health technologies seek to enhance the efficiency of healthcare provision by rapidly adjusting to healthcare settings to provide citizens with higher-quality medical services [5]. The precision of health information quality impacts hospital efficiency [6]. The computerized health information system HIS is essential for hospital operations as it assists decision-makers in diagnosing situations and streamlines the delivery of high-quality healthcare services, eventually leading to patient satisfaction [7]. Additionally, electronic health records EHRs have become a prominent and valuable source of real-world data due to the widespread digitalization of medical information worldwide. The data contained in EHRs encompasses a diverse range of patient information and healthcare process data derived from real-world settings, capturing their focus and attention [8].

Moreover, previous research has established that the degree of quality frequently corresponds to the prevailing infrastructure, state-of-the-art technology, and the equipment and activities performed by the workforce [9]. However, the provision of a high-quality health service is contingent upon various additional factors, such as the expertise and competence of the medical personnel, their willingness to support patients, their ability to pay attention to patient needs, the presence of adequate physical facilities, equipment, and communication channels, and an organizational culture that is supported by effective management [10]. Despite initial projections, their rapid acceptance has led to health inequities, including differences in health status and unequal allocation of healthcare resources across different groups [5]. Efficient health data administration is crucial for minimizing medical errors, improving operational efficiency, and enabling decision-making [11].

Strategic foresight is a systematic process based on quantitative or qualitative analysis that offers a useful chance to investigate possible future scenarios. While insight into government cannot dictate policy, it can help alter policies to be more relevant, adaptable, and resilient over time [12]. During rapid times of change, it is important to understand how the “past, present, and future” are interconnected, to take advantage of uncertainty, and to make responsible policy decisions that involve forecasting, strategic management, and the identification of future strategic opportunities [13]. Moreover, strategic foresight for the future of health promotion in the fast-changing 21st century aims to discover current analysis of health system capacity, foster proactive vision, inform future planning for those supporting the Sustainable Development Goals, and use scenarios to understand better possible futures and the proactive response process [14, 15]. Similarly, when considering the growing array of factors that affect work, the workplace, and the workforce, it is necessary to adopt innovative approaches to Occupational Safety and Health OSH in research and implementation. Consequently, this field employs strategic foresight to enhance its ability to anticipate and shape future developments. It involves creating and evaluating realistic scenarios that predict likely future outcomes, which then guide strategic decision-making [16].

Healthcare technology development faces challenges due to a lack of guidelines, financial constraints, privacy concerns, technological constraints, infrastructure limitations, data estimation issues, administrative obstacles, and the complexity of the healthcare sector, potentially reducing patient satisfaction and work opportunities [17].

Although Jordanians have access to a diverse range of healthcare facilities, many often perceive the overall quality of these services as subpar, especially in government-run health hospitals [18], resulting in disparities in care accessibility [19]. Various factors, such as the pressure refugees place on the healthcare system, contribute to the decline in the quality of healthcare services in Jordan, this has led to a need for prioritizing essential healthcare services and reducing the financial burden on patients [18]. Furthermore, telehealth technologies in government health facilities are insufficient to reduce the need for in-person visits for certain healthcare services. Furthermore, the failure to adjust to modern technologies that simulate real-life experiences in the healthcare industry can hurt the quality of patient care and lead to delays in diagnosis, treatment, accurate monitoring, resource utilization, time management, and decision-making speed [18, 19].

The Arab NGO Network [18] suggested the creation of a national health information infrastructure that guarantees convenient access to information from different fields and regulatory laws. This infrastructure should involve all sub-sectors and official institutions to facilitate exchange and enhance the use of electronic health technologies. The ultimate goal is to alleviate overcrowding in healthcare facilities and enhance patient care.

The authors propose using improved metrics, such as health outcomes, public trust, system efficiency, and user experience, to evaluate healthcare systems at local and national levels [20]. They also suggest reevaluating our strategies to demand and invest more in these crucial health factors; therefore, it is imperative to heed the researchers’ suggestions and prioritize the improvement of computerization to advance HIS development and maximize its potential benefits [11, 20].

Implementation issues have impeded the timely improvement of the systematic administrative, strategic systems designed to meet the fundamental national goals [11]. Therefore, Policymakers realize that they need to set rules and guidelines to deal with the growing use of AI-powered solutions in areas like society, ethics, the economy, and security [21, 22]. While AI can offer valuable services to stakeholders, it is important to acknowledge that Jordan, as a developing country, has not fully embraced AI technology in its health system [23].

After reviewing all of the literature above, we see a knowledge gap in investigating the significance of AI-based solution adoption, which is a subfield of information technology, in healthcare quality in governmental hospitals in Jordan. This research is considered to be cutting-edge in the sector. Additionally, there is a lack of research, both in the Arabic region and globally, that investigates the significance of strategic foresight and the presence of foresight experts to improve the quality of healthcare service in governmental hospitals in Jordan. This is a new topic in this scope, and limited research has been conducted on it. Furthermore, according to the researcher’s knowledge after reviewing the previous studies, there is a knowledge gap in the literature to identify the strength and direction of the link between the three variables and each other, particularly in the health sector and elsewhere. In addition, previous research did not investigate how an AI-based solution mediates the connection between strategic foresight and the quality of healthcare services.

This research uses a descriptive quantitative-analytical, cross-sectional correlational approach to examine the impact of strategic foresight on the quality of healthcare services provided by Jordanian nurses in the context of artificial intelligence (AI) solutions.

### Objective and research questions

The study’s significance arises from the hazards in the current healthcare environment, which has led numerous health organizations to devise novel strategies to cater to the needs of patients in various settings within the ever-evolving and uncertain context of the continuously growing healthcare services sectors. Additionally, this study can enhance patient-centered care by augmenting nurses’ understanding of patients’ evolving needs, values, perspectives, and preferences. Patients’ active involvement in collaborative decision-making, consideration of cultural nuances, and provision of appropriate education and support are paramount. Furthermore, improving patient safety and treatment outcomes in high-quality healthcare entails minimizing errors, harm, infections, and medication management.

The research questions we investigate are:

1. Does strategic foresight affect the quality of healthcare services among nurses in governmental hospitals in Jordan?
2. Does strategic foresight affect AI-based solution adoption among nurses in governmental hospitals in Jordan?
3. Does AI-based solution adoption affect the quality of healthcare services among nurses in governmental hospitals in Jordan?
4. Does the adoption of artificial intelligence AI-based solutions mediate the relationship between strategic foresight and the quality of healthcare services among nurses in governmental hospitals in Jordan?

## Methods

### Design and setting

This study conducted a descriptive quantitative-analytical, cross-sectional correlational approach. Compared to other research methods, cross-sectional studies control cost and time and provide the first evidence for advanced study planning, making it difficult to determine the direction of connections between variables or infer causality [24].

The study settings represent governmental hospitals registered and affiliated with the Jordanian Ministry of Health (JMOH) in the central region (Amman, Salt, Zarqa, and Madaba) as the main governmental comprehensive and transformational hospitals that provide secondary and tertiary health care in Jordan. Hospitals have been selected based on having the highest number of beds in each governorate. Four hospitals were approved (Al-Basheer Hospitals, New Zarqa Governmental Hospital, Al-Hussein Hospital, and AL-Nadeem Hospital) as these hospitals are the largest in terms of bed capacity and the most representative in terms of the highest population density in each governorate, with a total of 2868 nurses according to [25].

Nurses are the largest healthcare professionals who are the first and most common contacts for people of all backgrounds seeking medical care [26]. Therefore, the study sample included nurses working in hospitals. A convenient sampling method was employed. Given the demands of participants’ schedules and workload, it streamlines the process of identifying, registering, and inviting participants and recruiting qualified and available volunteers [27].

### Sampling

The study sample was calculated according to the number of nurses using G*Power software, which was created as a general stand-alone power analysis application for statistical tests frequently used in social, behavioral, and biomedical sciences [28]. Using a power estimate of 0.8, an alpha set at (0.05), a medium effect size of (0.15), and 8 predictors. With the convenience sampling strategy, participants were selected depending on their accessibility and availabilities during data collection. The needed sample size was approximately (109). We collect a larger sample size (270). As the sample size increases, the distributions narrow, reducing the probability of a Type II error [29].

Other medical and non-healthcare workers have been excluded. Inactive nurses—on leave, retired, or not practicing—are also excluded. The National Center for Mental Health and its affiliates (Al Karama Hospital and Addicts Rehabilitation Hospital) were excluded due to the lack of the number of nurses detailed in the report [25]. Furthermore, the institution has a specialization in the field of psychology.

### The study measurements

The researcher used Google Forms to create a five-point Likert scale and self-reported questionnaire. The questionnaire was translated into Arabic to enhance participant involvement after being prepared for the study environment. After translating the Arabic version, an English translation expert back-translated it to assure compatibility and no deviation. Which is divided into (A) Strategic foresight has [19] items. Cronbach’s alpha was around 0.7, showing internal consistency according to [30]. (B) Quality of healthcare services is the dependent variable with [20] items based on the SERVQUAL model with a Cronbach alpha value of over 0.7, according to [31]. (C) AI-based solutions consist of [15] items based on TAM, with a Cronbach alpha value of over 0.70, according to [32], with a total of 54 items.

Therefore, the researcher recalculated internal consistency coefficients for all questionnaire items using Cronbach’s alpha method to maintain the questionnaire’s reliability. The Cronbach alpha values for each strategic foresight, AI-based solutions adoption, and the quality of healthcare services were 0.960, 0.941, and 0.959, respectively. The study scale’s internal consistency and reliability were excellent. Thus, the items measured in the study tool strongly correlate.

**Table 1:** Supporting reliability table

No.	Variable	Coefficient of Cronbach’s Alpha	Reliability Level
1	Strategic Foresight	0.96	Excellent
2	AI-Based Solutions Adoption	0.94	Excellent
3	The Quality Of Healthcare Services	0.95	Excellent

### Data collection

After preparing the research plan for the study in its final form by verifying the validity and reliability of the tools and designing the questionnaire by using Google Forms, in line with what was mentioned above, the researcher explained the importance of the study to nurses and their participation in it, that their participation was voluntary, that they could withdraw from it freely and without any penalties, and that it would take 15 to 20 min to fill out. The data collection process took three weeks between January 2024 and February 2024. The distributed questionnaires amounted to (270), (240) were retrieved and were valid for analysis after processing the missing data and outliers to be fully complete, consistent, and accurate according to the SPSS program, representing 88.9% of the sample.

### Data analysis

The questionnaire data were analyzed using the Statistical Package for the Social Sciences (SPSS) v26 and AMOS SPSS v26. Appropriate statistical tests were used to answer the study questions.

### Ethical considerations

Concerned approvals were obtained from relevant authorities to facilitate the ethical study protocol with IRB approval No. 3/2023.

It should be noted that the Office of Continuing Education in each hospital was reviewed, ethical pledges were signed, and a copy of the questionnaire was reviewed before starting in a documented and legal manner.

Moreover, the tools for the three variables were used after obtaining the approval of their authors and after ensuring their validity and reliability. In addition, approval was taken from study participants, and there is no funding for this study.

## Results

Table 1 provides the demographic features of the sample under examination. The data is divided into three categories: gender, educational level, and nurses’ jobs, with extra information on age and years of experience. Regarding gender distribution, the sample is 80.4% female and 19.6% male. This gender breakdown indicates a majority of females in the sample. According to educational level data, the majority of participants (59.6%) have a bachelor’s degree, followed by those with a Diploma degree (30.8%) and a Postgraduate degree (9.6%). The nurse job category represents the various roles in the nursing profession. Registered nurses (64.6%) make up the largest category, followed by registered midwives (25.8%), associate nurses (7.9%), and a modest proportion of nursing Assistants (1.7%).

**Table 2:** Distribution of the studied nurses according to demographic data (*n* = 240)

Variable		Frequency	Percent
Gender	Female	193	80.4
	Male	47	19.6
	**Total**	**240**	**100.0**
Educational Level	Diploma degree	74	30.8
	Bachelor’s degree	143	59.6
	Postgraduate degree	23	9.6
	**Total**	**240**	**100.0**
Nurses Job	Registered Nurse	155	64.6
	Associate Nurse	19	7.9
	Registered Midwife	62	25.8
	Nursing Assistant	4	1.7
	**Total**	**240**	**100.0**
	**Average Std. Deviation**		
Age	36.16 ± 7.941 7.9408		
Year of Experience	12.73 ± 7.937 7.9369		

Correlation coefficient (Pearson’s r) and simple linear regression have been used to answer the study questions. Table (2) shows a correlation coefficient (r) of 0.279 between strategic foresight and the quality of healthcare services, 0.543 between strategic foresight and the adoption of AI-based solutions, and 0.432 between the adoption of AI-based solutions and the quality of healthcare services; the correlation is statistically significant at the 0.01 level. The positive correlation coefficient indicates a linear relationship between the variables.

Simple linear regression results reveal that the F-statistic of (20.079) is highly significant with a p-value (0.001), which is less than (0.05) with (*R* = .279, R square = 0.078), which means that 7.8% of the change in the quality of health services can be explained by strategic foresight. Also, the F-statistic of (99.442) is highly significant with a p-value (0.001), which is less than (0.05) with (*R* = .543, R square = 0.295), which means that 29.5% of the change in AI-based solutions adoption can be explained by strategic foresight. The coefficient for strategic foresight is 0.354. Finally, results show that the F-statistic of (54.656) is highly significant with a p-value (0.001), which is less than (0.05) with (*R* = .432, R square = 0.187), which means that 18.7% of the change in the quality of healthcare services can be explained by AI-based solutions adoption.

**Table 3:** Correlation coefficients (Pearson’s R) and simple Linear regression

Model			*R*	*R* squared		Unstandardized Coefficients			Standardized Coefficients		T		sig		F	sig
						B		Std. Error	Beta							
(Constant)			0.279	0.078		71.070		3.307			21.488		0.000		20.079	0.001
Strategic foresight						0.223		0.050	0.279		4.481		0.000			
**Pearson Correlation (r)**				**Quality of healthcare services**												
**Strategic foresight**				0.279**												
**Model**	**R**			**R squared**	**Unstandardized Coefficients**					**Standardized Coefficients**		**T**		**sig**	**F**	**sig**
					**B**		**Std. Error**			**Beta**						
(Constant)	0.543			0.295	34.297		2.354					14.570		0.000	99.442	0.001
Strategic foresight					0.354		0.035			0.543		9.972		0.000		
**Pearson Correlation (r)**				**(AI)- Based solutions adoption**												
**Strategic foresight**				0.543^**^												
**Model**		**R**		**R squared**	**Unstandardized Coefficients**					**Standardized Coefficients**		**T**		**sig**	**F**	**sig**
					**B**		**Std. Error**			**Beta**						
(Constant)		0.432		0.187	55.180		4.164					13.251		0.000	54.565	0.001
(AI)- Based solutions adoption					0.531		0.072			0.432		7.387		0.000		
**Pearson Correlation (r)**				**Quality of healthcare services**												
**(AI)- Based solutions adoption**				0.432^**^												
**. Correlation is significant at the 0.01 level (2-tailed).																

**Fig. 1:**
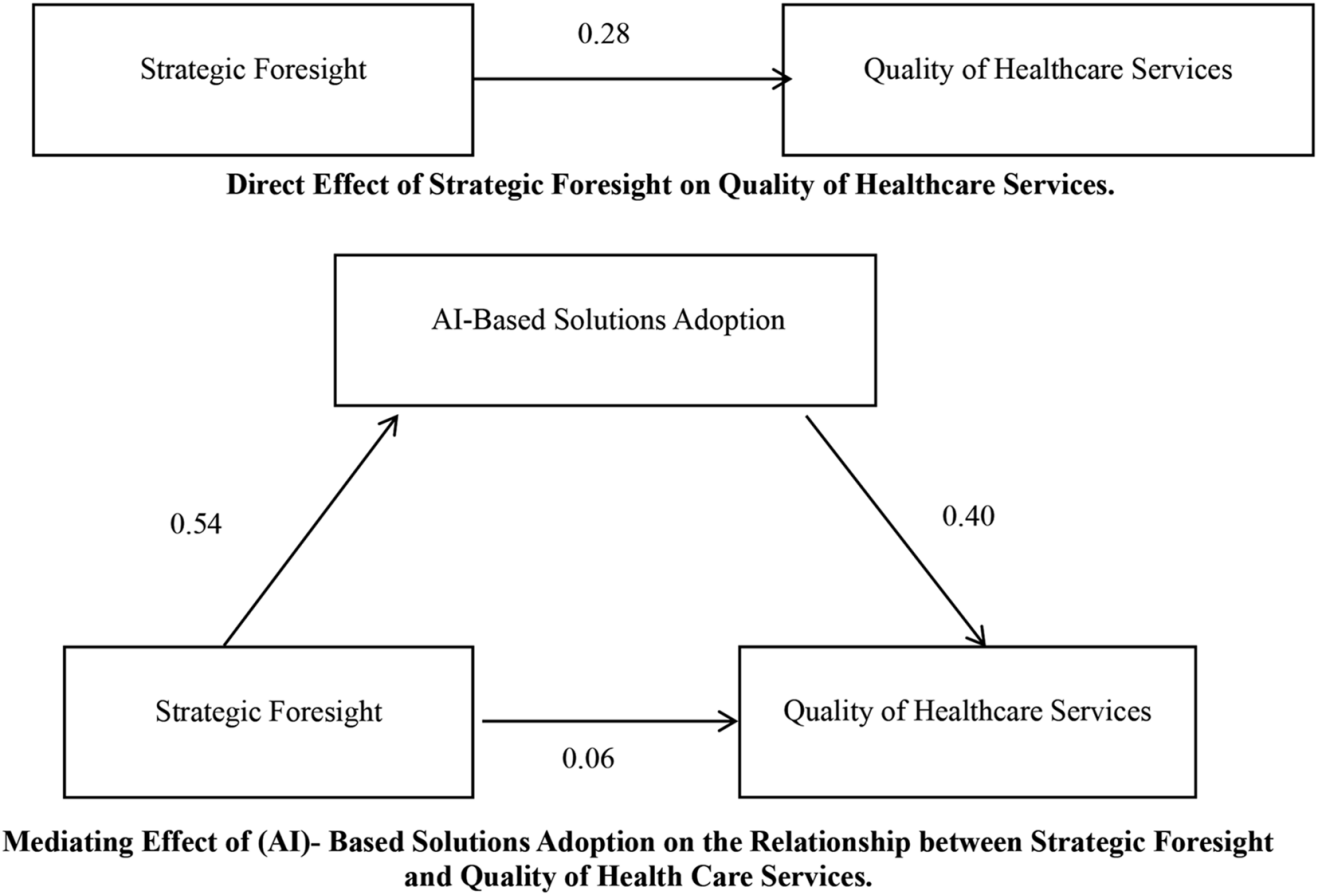
Direct and Indirect Effect (Developed by Alajrab et al. 2024 ©)

Table (3) shows a positive and significant indirect effect of strategic foresight on the quality of healthcare services (b = 0.216, *p* = .004); the study revealed that the direct effect of strategic foresight on the quality of healthcare services, in the presence of the mediator, was not statistically significant (b = 0.063, *p* = .398). Therefore, adopting AI-based solutions completely mediates the relationship between strategic foresight and the quality of healthcare services. This implies that strategic foresight has influenced the quality of healthcare services through an intermediary. The results also show that the first model, which revealed that strategic foresight has a significant effect on the quality of healthcare services (standardized β = 0.28, *p* < .01), as shown in Fig. (1) (See Table 4).

**Table 4:** Path coefficients

Path	Standardized Beta		Standard error		*p*-value
Strategic Foresight –< Quality of healthcare services	0.28		0.050		0.001
**Path**	**Direct Effect**	**Indirect Effect**	**Confidence Interval**		**Conclusion**
Strategic Foresight –< AI-based solutions–< and Quality of health care services	0.063 (p-value = 0.398)	0.216 (p-value = 0.004)	**Lower Bound**	**Upper Bound**	Complete Mediation
			0.076	0.269	

## Discussion

The study found a clear connection between strategic foresight and the quality of healthcare services among nurses in governmental hospitals in Jordan. Our study results support the findings of [33], who utilized a participatory foresight approach to examine and develop a model for implementing eHealth services for informal dementia caregivers in Ontario. This has been accomplished by understanding the needs of caregivers, the impact of advancing technologies, and the factors that affect the implementation of eHealth solutions. In addition, our results support the findings of the [34] study, highlighting a strong connection between strategic foresight and cost-saving strategies, ultimately enhancing the quality of service.

In the same vein, our study results support the findings of the results of a recent study by [35]; their research also demonstrated a significant impact of strategic thinking on organizational excellence, particularly when strategic foresight is considered. This correlation is further enhanced by strategic foresight, which directly contributes to the overall quality of service. In line with the findings of [30], our study also highlights the importance of fostering strategic discourse within businesses. This involves engaging all relevant parties, focusing on the employees, and helping achieve the organization’s goals that demonstrate high-quality service.

The results showed a statistically significant effect between adopting strategic foresight and AI-based solutions. Our study results support the findings of [35], which demonstrated the accurate and efficient prediction capabilities of the proposed AI-based solutions methods. It was also proposed that strategic foresight and AI-based solutions modeling can help predict future agility. Additionally, our study results support the findings conducted by [36], which showed a strong link between the adoption of AI technology and the emergence of new job prospects that positively affect the human aspect. Our study results support the findings of [1], which revealed that using AI-based solutions in strategic foresight is currently limited but holds promising potential. Moreover, our study results support the findings of [32] in AI-based solutions in healthcare projects. It is recommended that researchers consider external factors influenced by their organizational culture and operations and implement best practices to ensure project success.

The results revealed a statistically significant effect between adopting AI-based solutions and healthcare service quality. The results of our study support the findings of the research conducted by [37], suggesting that various components of AI, such as expert systems, genetic algorithms, neural networks, and smart systems, substantially impact healthcare facilities’ decision-making capabilities. In addition, our study results support the findings of a study conducted by [38] in western Sweden, which provided recommendations for enhancing innovation in the healthcare system. These strategies involve political efforts to enhance resources, visibility, and mission for utilizing AI-based solution technology in healthcare and promote awareness of system dynamics and interconnections.

In addition, our study results support the research findings [39], highlighting the ability of generative models AI to analyze sensor data and offer personalized medical recommendations. This has the potential to enhance the delivery of individualized care, improve patient outcomes, minimize trial and error in treatment selection, and simplify healthcare delivery.

Also, our study findings support the findings of [40], highlighting the significant value of the “5G plus AI” combination in various areas, including initial discovery, monitoring and early warning, diagnosis aid, pathology sharing, and diagnosis and treatment development. In addition, AI can greatly decrease the risk of cross-infection and treatment burden, resulting in enhanced treatment efficiency. In addition, our study findings support the research conducted by [6] in Jordan. Their study revealed a significant and interconnected relationship between HIS, hospital performance, and health information quality, highlighting their positive impact on each other. Furthermore, our study findings support the findings of a study conducted by [7] in Iraq. The research demonstrated a positive relationship between using a Computerized HIS and improving healthcare services in hospitals.

The result showed that statistically significant adoption of AI-based solutions completely mediates the relationship between strategic foresight and the quality of healthcare services among nurses in Jordan. This is linked to strategic foresight theory, the SERVQUAL Model, and the TAM. By integrating these models, healthcare services can be strengthened sustainably, improving quality and patient participation. AI can be used for big data analysis, predicting future trends, and ensuring the acceptability and implementation of health technology. This approach improves patient experience and satisfaction through its adoption and successful utilization. It prioritizes patient needs and adapts to changes in the health sector.

### Implications

Adopting AI solutions based on strategic foresight has tangible benefits for early disease detection, accurate diagnosis, and effective treatments, resulting in improved health outcomes. Additionally, it can improve public health by recognizing healthcare needs and forecasting changes in population demographics.

Strategic foresight enables nurses and nursing organizations to predict and prepare for population developments, adapt methods, and acquire specialized expertise.

This study is significant for effectively controlling patient volumes, mitigating overcrowding, reducing wait times, guaranteeing an adequate number of qualified nurses, and enhancing patient-centered care. AI solutions with strategic foresight have significant policy implications as they aid in strategic planning by detecting future scenarios and trends and effectively addressing issues and opportunities in the healthcare sector.

Additionally, it aids in tackling issues encountered in government hospitals, such as predicting and allocating resources, controlling staff, and enhancing technology proficiency.

### Recommendations

**Fig. 2:**
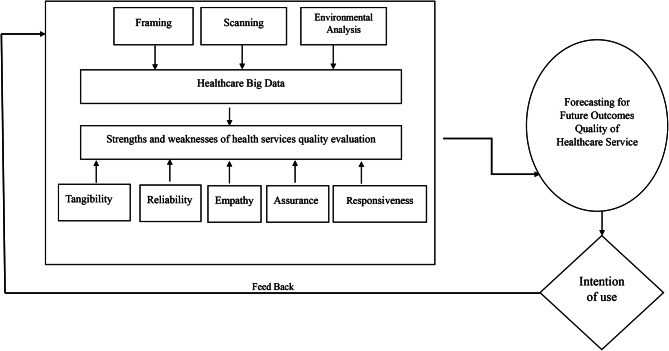
A simulation proposed model for a healthcare quality forecasting system based on (AI)

The researcher recommends merging the technology acceptance model, SERVQUAL theory framework, and strategic foresight framework to design utilizing AI systems (A simulated suggested framework for a healthcare quality forecasting system) according to the proposed model created by the author in Fig. 2.

The study results suggest nurse managers and change facilitators in hospitals should prioritize thorough analyses of the underlying causes of challenges that prevent the adoption and implementation of AI technologies in healthcare sectors. Furthermore, future research should prioritize investigating the obstacles and factors that can impact the ability to forecast the quality of healthcare service outcomes, considering both the perspectives of patients and healthcare providers.

It also enhances healthcare quality by fostering a culture of participation among managers and nurses, enhancing their involvement in strategic decision-making processes.

In addition, hospital administrators should prioritize the development of long-term strategic plans in healthcare by leveraging AI technologies for anticipation and prediction.

This study recommends that healthcare professionals adjust to evolving policies and systems by enhancing their abilities and integrating feedback into decision-making. Facilitating cooperation among hospitals and other institutions to exchange information and expertise will thus improve their ability to adapt to rapid changes in the healthcare environment.

The study additionally offers recommendations for future research directions, such as analyzing factors that influence nursing behavior in hospitals, evaluating nurse experiences with digital health systems, and analyzing healthcare quality and its relationship to strategic foresight and AI adoption.

Finally, future research should explore barriers and facilitators factors at patients, nurses, and healthcare provider levels that influence health service quality outcome prediction.

### Limitations

The study had several limitations as it used a non-experimental descriptive methodology, which limits its ability to prove causality and correlations. Also, a cross-sectional approach involved measuring critical variables at a single time rather than repeatedly over time. Moreover, sampling was non-random, with research settings selected based on specific hospitals, restricting the generalizability of the findings to nurses and hospitals with similar characteristics. The study utilized self-report questionnaires that relied on respondents’ information retention.

## Conclusions

Based on the results of this study, strategic foresight is directly and significantly related to the perceived quality of healthcare services and the adoption of AI-based solutions in governmental hospitals. Also, AI-based solutions significantly and indirectly mediated the relationship between strategic foresight and the perceived quality of healthcare services. Therefore, nursing administrators are recommended to facilitate the incorporation of strategic insight and AI-based solutions to improve healthcare services.

## Data Availability

Data are available upon request.
